# Study on influence of external factors on the electrical excitability of PC12 quasi-neuronal networks through Voltage Threshold Measurement Method

**DOI:** 10.1371/journal.pone.0265078

**Published:** 2022-03-09

**Authors:** Xiao-Ying Lü, Chen Meng, Shuai An, Yong-Fang Zhao, Zhi-Gong Wang

**Affiliations:** 1 State Key Laboratory of Bioelectronics, Southeast University, Nanjing, Jiangsu Province, China; 2 Co-innovation Center of Neuroregeneration, Nantong University, Nantong, Jiangsu Province, China; 3 Institute of RF- & OE-ICs, Southeast University, Nanjing, Jiangsu Province, China; The University of Texas Rio Grande Valley, UNITED STATES

## Abstract

The aim of this paper was to investigate the influence of four different external factors (acetylcholine, ethanol, temperature and lidocaine hydrochloride) on PC12 quasi-neuronal networks by multielectrode-array-based Voltage Threshold Measurement Method (VTMM). At first, VTMM was employed to measure the lowest amplitude of the voltage stimulating pulses that could just trigger the action potential from PC12 quasi-neuronal networks under normal conditions, and the amplitude was defined as the normal voltage threshold (*V*_Th_). Then the changes of the *V*_Th_ of PC12 quasi-neuronal networks treated by the four external factors were tested respectively. The results showed the normal *V*_Th_ of PC12 quasi-neuronal networks was 36 mV. The *V*_Th_ has negative correlation with the concentration of acetylcholine and has positive correlation with the concentration of ethanol. The curves of the correlation of the *V*_Th_ with temperature and the concentration of lidocaine hydrochloride were U-shaped and Λ-shaped respectively. Comparing with our earlier studies on hippocampal neuronal networks and hippocampal slices, PC12 quasi-neuronal networks not only had the same typical voltage threshold characteristic, but also had similar changes on electrical excitability when treated by the four external factors mentioned above. Therefore, the rapid-formed PC12 quasi-neuronal networks could replace neuronal networks in proper conditions, and VTMM could be used to analyze the influence of external factors on the electrical excitability of PC12 quasi-neuronal networks.

## Introduction

In 1972, Charles A. Thomas Jr. invented microelectrode array (MEA), which could be used to measure the electrical activities of neuronal networks cultured on its surface and implemented patch clamp’s limitation that the electrophysiological characteristics of only one single neuron could be tested each time [1]. After half a century, scientists have developed multiple types of MEAs and established neuron culturing techniques on MEA to investigate neuronal networks’ electrophysiological characteristics, such as spontaneous and triggered action potentials [2–5]. While limited by the traditional data analysis methods, most of the MEA experiments required long recording time and complicated statistical computation. In order to study the electrophysiological characteristics of neuronal networks in an easier and faster way, our group has developed the Voltage Threshold Measurement Method (VTMM) based on MEA techniques [6]. Using the voltage threshold principle of voltage clamp during single neuron action potential measurement for reference, VTMM can quantitatively study the influence of different factors on the electrical excitability of neuronal networks by finding out the voltage threshold (*V*_Th_) which could trigger the responses from the networks in resting status and testing the changes of the *V*_Th_ under the effect of applied factors. The principle of VTMM is clear, and VTMM also has the advantages of an easy and time-saving experiment procedure and straightforward results. However, during experiments, we found that the process to acquire neuronal networks through primary hippocampal neuron culture or hippocampal slices was sophisticated and it was not in accord with the 3R (Reduction, Refinement and Replacement) principles for animal protection. Therefore, it is valuable to find a simple network model as a substitute for normal neuronal networks for VTMM and studying the electrophysiological characteristics of neuronal networks in an easier and faster way.

Differentiated rat adrenal pheochromocytoma (PC12) cells have the similar physiological and biochemical functions as sympathetic ganglion neurons [7]. It has been widely used in neurotoxicity, neuropharmacology and biocompatibility studies [8–10]. A series of studies have been completed through patch clamp and other methods to study the electrophysiological characteristics of PC12 cells, such as the responses to neurotransmitters and the types of ion channels [11, 12]. These studies have proved that PC12 cells share many electrophysiological features with neurons. Some MEA-based studies on the release of neurotransmitters of PC12 cells have been reported [13–15], and a study on the action potentials of PC12 cells has just been published recently [16]. However, no research has been performed to compare the electrical excitability of PC12 quasi-neuronal networks with that of normal neuronal networks yet. The objective of this paper is to investigate the effects of acetylcholine (ACh), ethanol, temperature and lidocaine hydrochloride (LDH) on *V*_Th_ of PC12 quasi-neuronal networks through VTMM, respectively, and further to compare the results with that of our previous studies completed with hippocampal neuronal networks and hippocampal slices. The aim is to explore the feasibility of replacing neuronal networks with rapid-formed PC12 quasi-neuronal networks model in related studies to research the electrical excitability of networks.

## Materials and methods

### Measurement setup

The system setup for measuring the *V*_Th_ through VTMM has been detailed in our previous paper [6]. In brief, the MEA was fixed in an MEA holder (MEA1060, Multi Channel Systems, Germany). The MEA holder connected with a temperature controller (TC01, Multi Channel Systems, Germany) and a multi-channel front-end neural signal amplifier (Cerebus, USA). Amplified signals were recorded by a 128-channel neuro-signal processor (Cerebus, USA) and displayed both on a computer and an oscilloscope (Agilent 2024A, USA). The stimulation was generated by a voltage pulse generator (Agilent 33220A, USA).

### Culture of PC12 quasi-neuronal networks on MEA

MEA (60MEA100/10iR-Ti, Multi Channel Systems MCS GmbH, Germany) was first prepared according to following steps:

1. The MEA chamber was immersed in 75% ethanol for 30 minutes, then dried and sterilized by ultraviolet light in a laminar flow cabinet for 8 hours.
2. 0.1-mg/mL PLL solution (Sigma-Aldrich, U.S.) was added into the culture chamber of the MEA to completely immerge all electrodes.
3. The MEA was incubated with PLL solution for 24 hours at 37°C, 5% CO_2_ and saturated humidity.
4. The PLL solution was removed, and the MEA was rinsed with sterilized ultra-pure water and dried in a laminar flow cabinet.

The culture medium for PC12 was prepared with DMEM medium (Gibco, U.S.), 10% fetal bovine serum (Hyclone, U.S.), and 1% penicillin-streptomycin solution (Biological Industries, Israel).

To build PC12 quasi-neuronal networks, highly differentiated rat PC12 cells (Shanghai Cell Bank of Chinese Academy of Sciences) with quasi-neuronal features were seeded onto the surface of the prepared MEA at a cell density of 1×10^4^/cm^2^. Then, the MEA with the PC12 cells was placed in an incubator and cultured with the culture medium for approximate 3 days at 37°C, 5% CO_2_ and saturated humidity.

### Selection of stimulating and detecting electrodes on MEA

**Fig 1:**
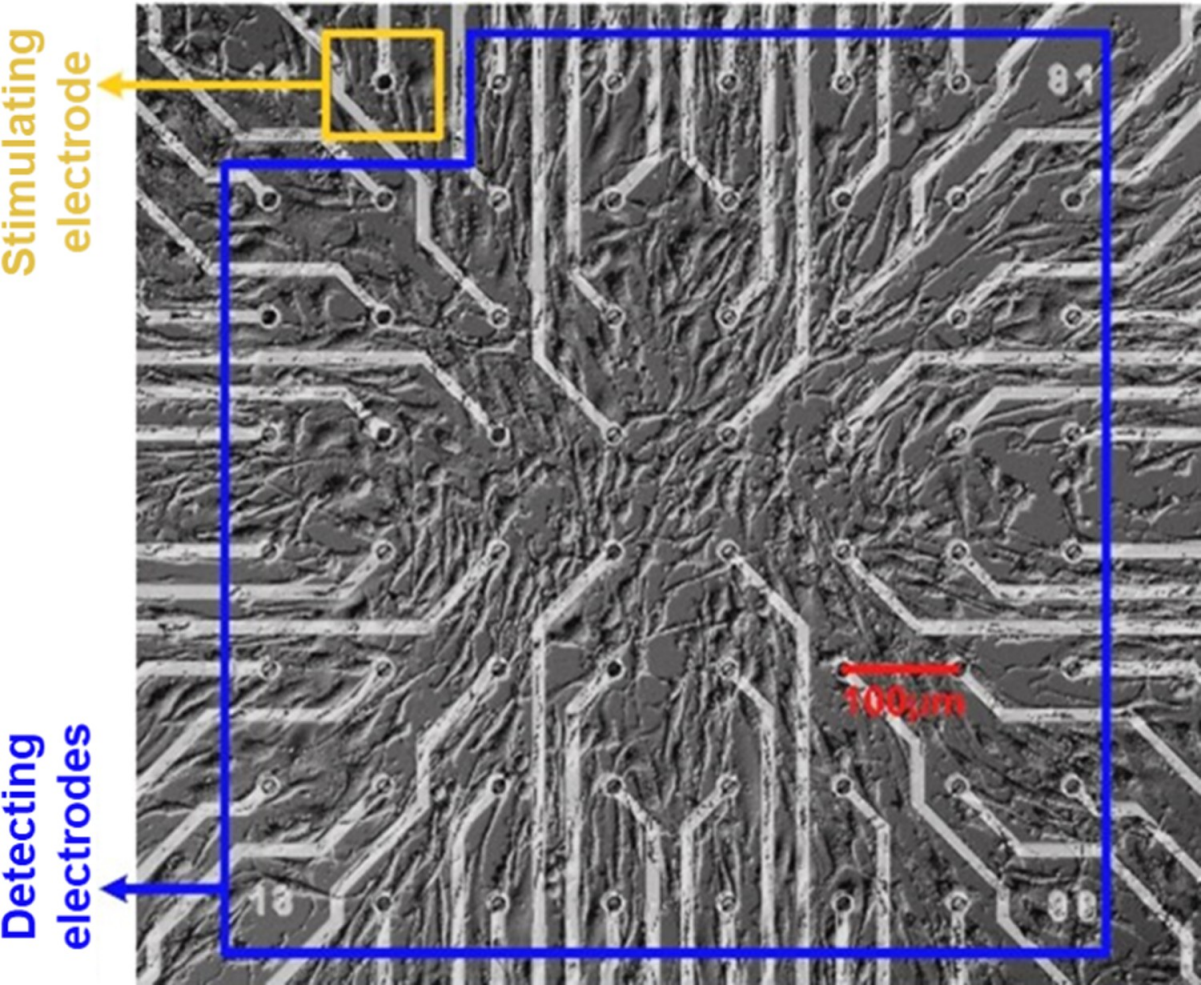
The status of PC12 quasi-neuronal networks on MEA and the selection of stimulating electrode (in yellow box) and detecting electrodes (in blue box).

As shown in Fig 1, the following experiments could be performed when the PC12 quasi-neuronal networks developed well and covered most part of the electrode area on the MEA. Meanwhile, an electrode covered by quasi-neuronal networks was selected as the stimulating electrode and all the other electrodes covered by the networks were set as the detecting electrodes.

### Measurement of normal *V*_Th_ and the *V*_Th_ under different influential factors

The PC12 quasi-neuronal networks cultured on MEA for 3 days was employed as the object of study. The experiment procedure and the selection of voltage stimulation waveform for measuring the normal *V*_Th_ through VTMM have been detailed in our previous paper [6]. In brief, voltage stimulation was used to trigger responses from the networks, and the neuro-signal processor and the oscilloscope were both used to supervise, recognize, and record the signals of triggered responses in real time. The lowest amplitude of the negative phase of the stimulation pulses which can trigger the responses from the networks at 37°C and with no external influential factors was defined as the normal *V*_Th_.

In experiments to investigate the effects of ACh, ethanol, and LDH on the *V*_Th_ of PC12 quasi-neuronal networks, the culture medium in the MEA chamber was replaced with fresh test medium containing a given concentration of one of the three chemicals. After 3 minutes of incubation, the *V*_Th_ of the networks was measured. Each MEA with PC12 quasi-neuronal networks would be used only once under one concentration of an influential factor for the *V*_Th_ measurement.

In experiments to study the influence of temperature on the *V*_Th_ of PC12 quasi-neuronal networks, the temperature was set at 37°C at first, and the changes of *V*_Th_ of the networks with the increase and decrease of temperature were tested separately with networks on different MEAs. The temperature was adjusted from 37–42°C or 37–33°C in 1°C steps. The *V*_Th_ of PC12 quasi-neuronal networks at a given temperature was measured after 3 minutes of incubation at the temperature.

In all experiments, the sample number was the number of MEA with neuronal networks used in the experiments.

### Data process and analysis

Shapiro-Wilk test was employed to test the normal distribution of the results from parallel groups through SPSS 20.0 software (IBM, Armonk, NY, USA). When the data were normally distributed (*P* > .05), we calculated the mean and standard deviations (mean ± SD) of test results from the parallel groups under the same conditions.

## Results

### Normal *V*_Th_ of PC12 quasi-neuronal networks

In experiments, the average normal *V*_Th_ of PC12 quasi-neuronal networks was 36 mV after 10 times of *V*_Th_ tests. According to the principle of VTMM and the definition of normal *V*_Th_, the influence of the applied external factors was inhibitory to PC12 quasi-neuronal networks when the *V*_Th_ was higher than the normal *V*_Th_ and was excitatory when the *V*_Th_ was lower than the normal *V*_Th_.

**Fig 2:**
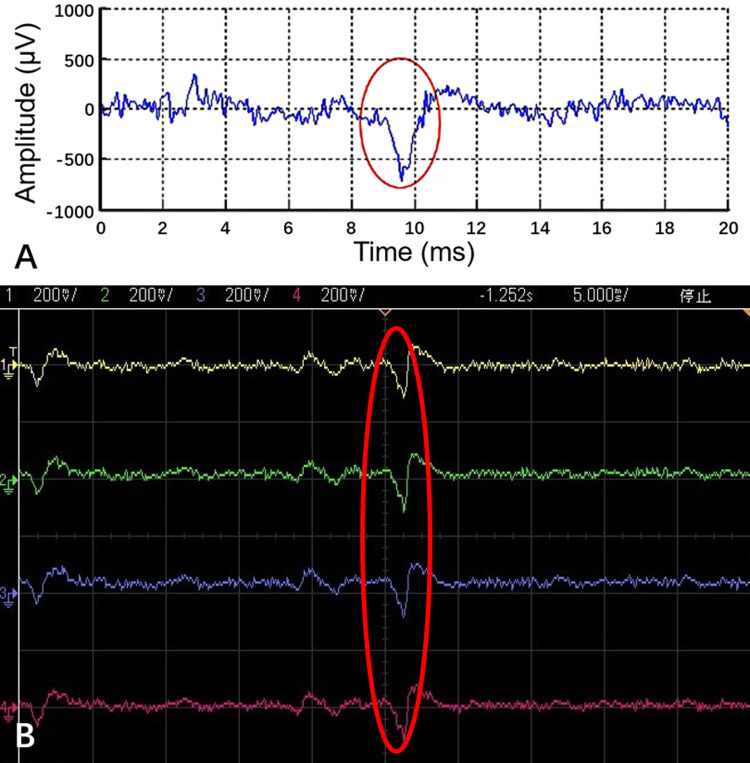
The signals of typical triggered responses of PC12 quasi-neuronal networks. A) The responses recorded by the neuro signal processor and the curve was plotted by MATLAB. B) The amplified responses recorded by 4 different channels were displayed on the oscilloscope. The peak-to-peak voltage of the stimulation was 40 mV at the time.

The signals recorded in the experiments (Fig 2) were uniform with the extracellular recorded action potentials of PC12 quasi-neuronal networks reported in another published study [16], thus it could be confirmed that the recorded signals were the signals of typical triggered responses and the results proved that PC12 quasi-neuronal networks could meet the requirement of VTMM. No research has clearly reported spontaneous electrical activities of PC12 quasi-neuronal networks at present, and no spontaneous signals from the networks were observed in the first 10 min during each test in our experiments.

### The effects of different factors on the *V*_Th_ of PC12 quasi-neuronal networks

**Fig 3:**
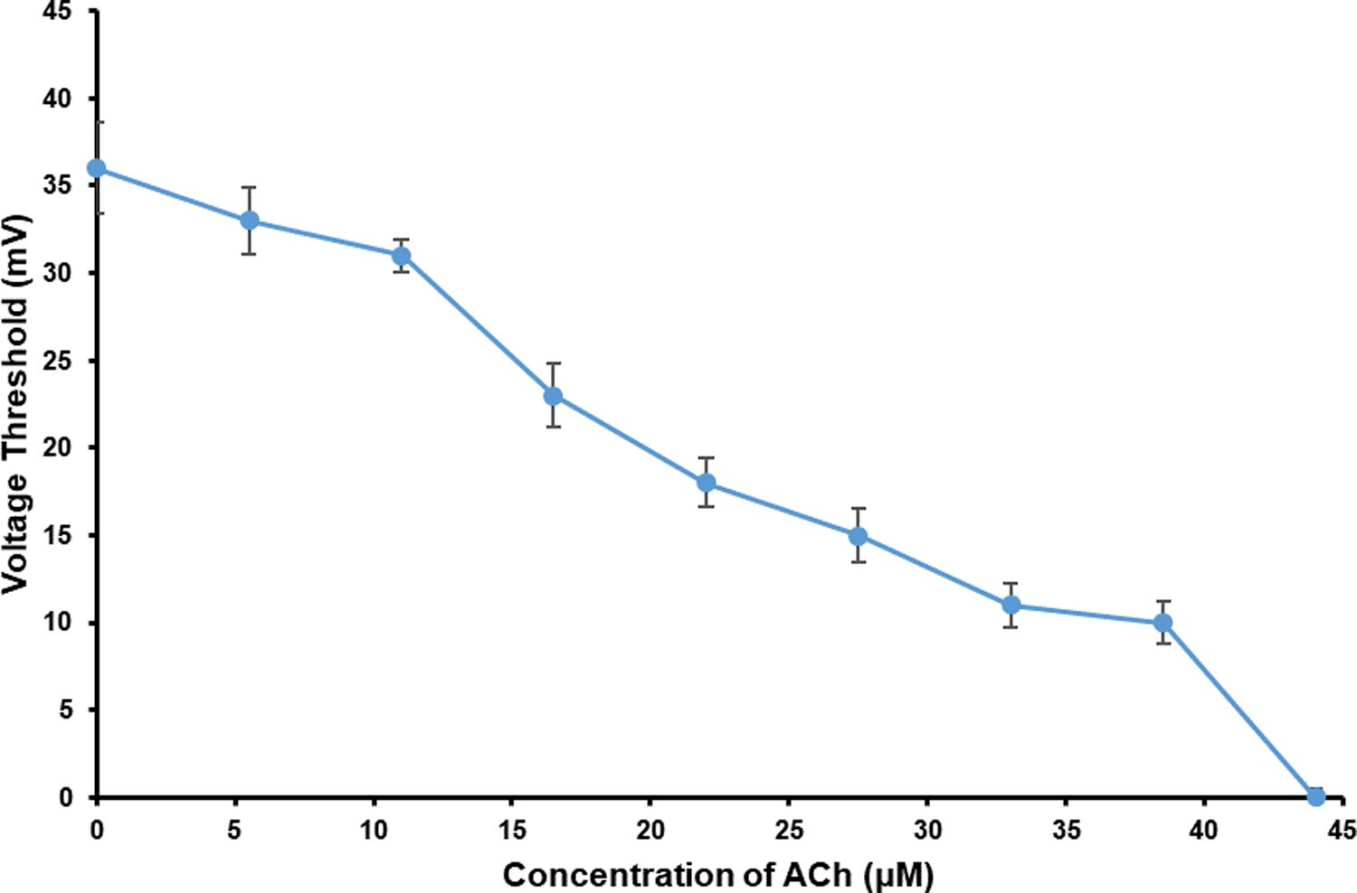
The *V*_Th_ of PC12 quasi-neuronal networks under the effect of ACh in different concentrations (*n* = 5).

As shown in Fig 3 and S1 Table, the average *V*_Th_ of PC12 quasi-neuronal networks went down with the increase of ACh concentration, which represented an increase in networks electrical excitability. When the concentration of ACh reached 44 μM, the *V*_Th_ dropped to 0 mV, which meant the PC12 quasi-neuronal networks could generate responding signals to ACh directly without electrical stimulation when the concentration of ACh ≥ 44 μM.

**Fig 4:**
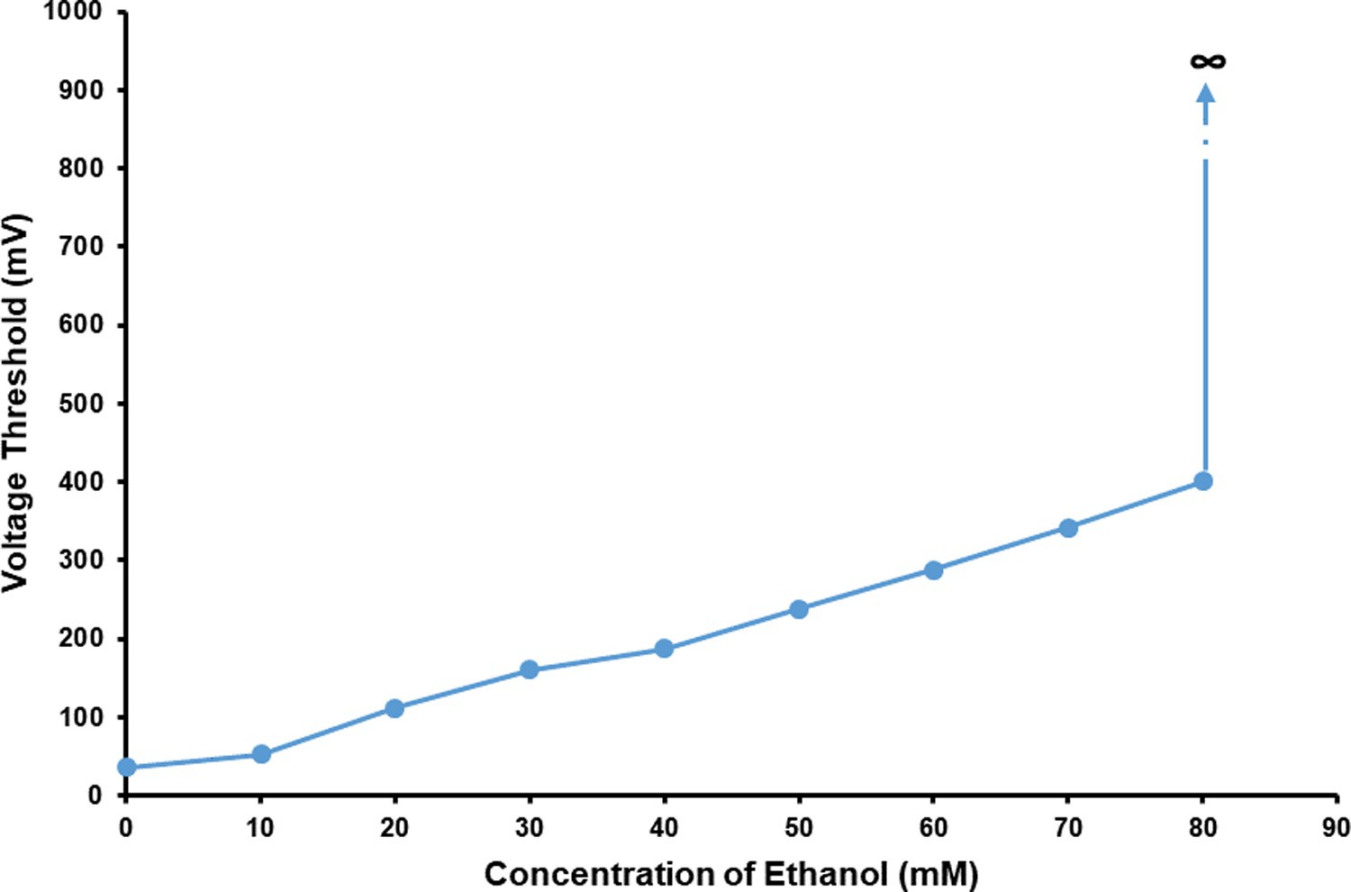
The *V*_Th_ of PC12 quasi-neuronal networks under the effect of ethanol in different concentrations (*n* = 5).

As shown in Fig 4 and S2 Table, the result of our experiments showed that the *V*_Th_ of PC12 quasi-neuronal networks went up with the increase of ethanol concentration, which proved the electrical excitability of the networks went down with the increase of ethanol concentration. When the concentration of ethanol reached or exceeded 80 mmol/L, no signals could be triggered from the networks despite further increasing the voltage amplitude of stimulation.

**Fig 5:**
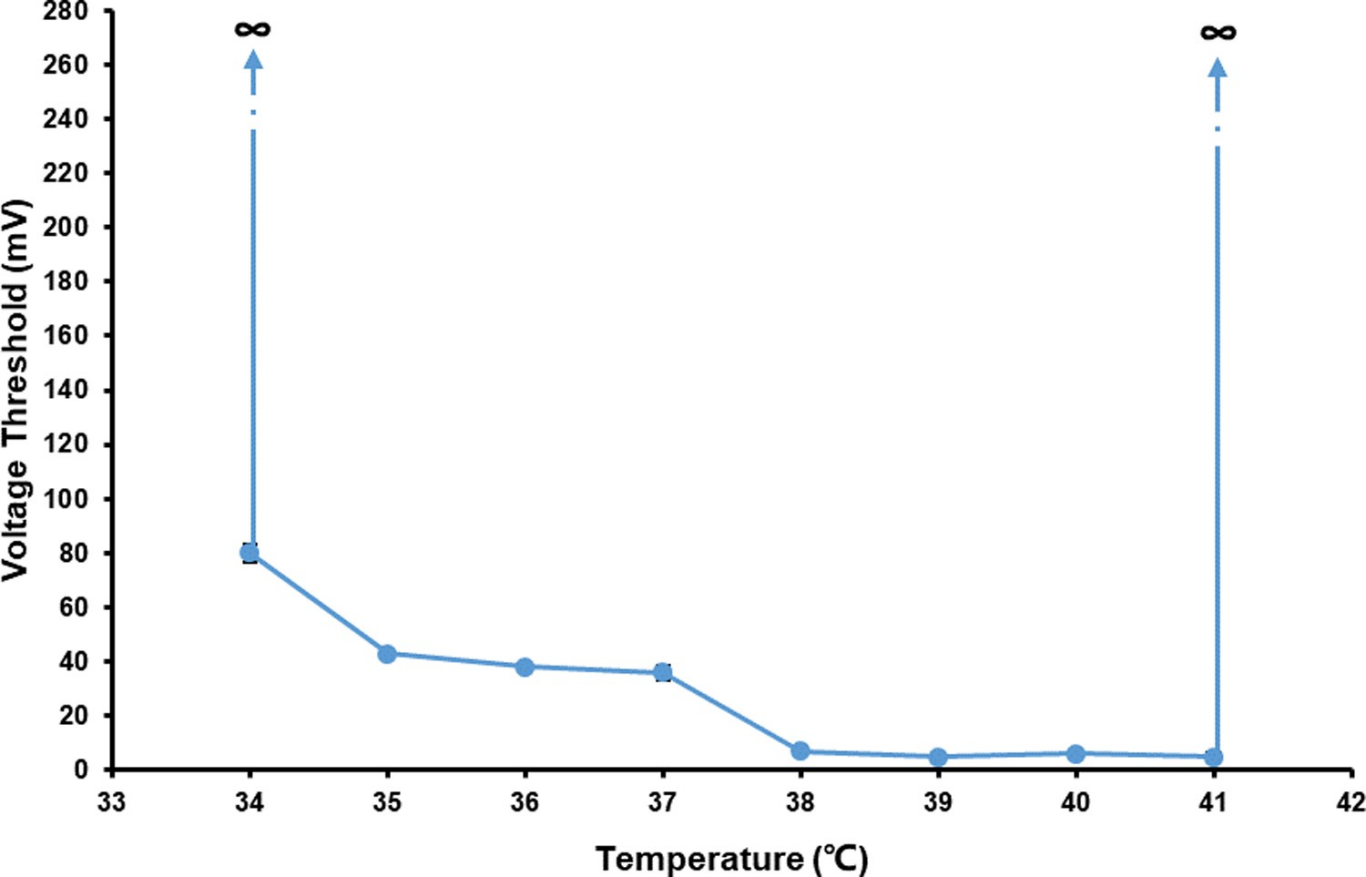
The changes of *V*_Th_ of PC12 quasi-neuronal networks with temperature (*n* = 5).

The whole curve of the changes of *V*_Th_ of PC12 quasi-neuronal networks with temperature was U-shaped (Fig 5, S3 Table). When the temperature increased from 37°C, the *V*_Th_ of PC12 quasi-neuronal networks went down first. But when the temperature rose to ≥ 42°C, the *V*_Th_ of PC12 quasi-neuronal networks had no responses to stimulations despite further increase of the stimulation voltage amplitude, which showed PC12 quasi-neuronal networks would lose its electrical excitability when the temperature was ≥ 42°C. When the temperature decreased from 37°C, the *V*_Th_ of PC12 quasi-neuronal networks went up. While the temperature went down to 33°C, PC12 quasi-neuronal networks lost its electrical excitability and further increase of the stimulation voltage amplitude could not trigger any responses. In the temperature range from 34 to 41°C, the *V*_Th_ has negative correlation with temperature. When the temperature was ≤ 33°C or ≥ 41°C, PC12 quasi-neuronal networks lost its electrical excitability.

**Fig 6:**
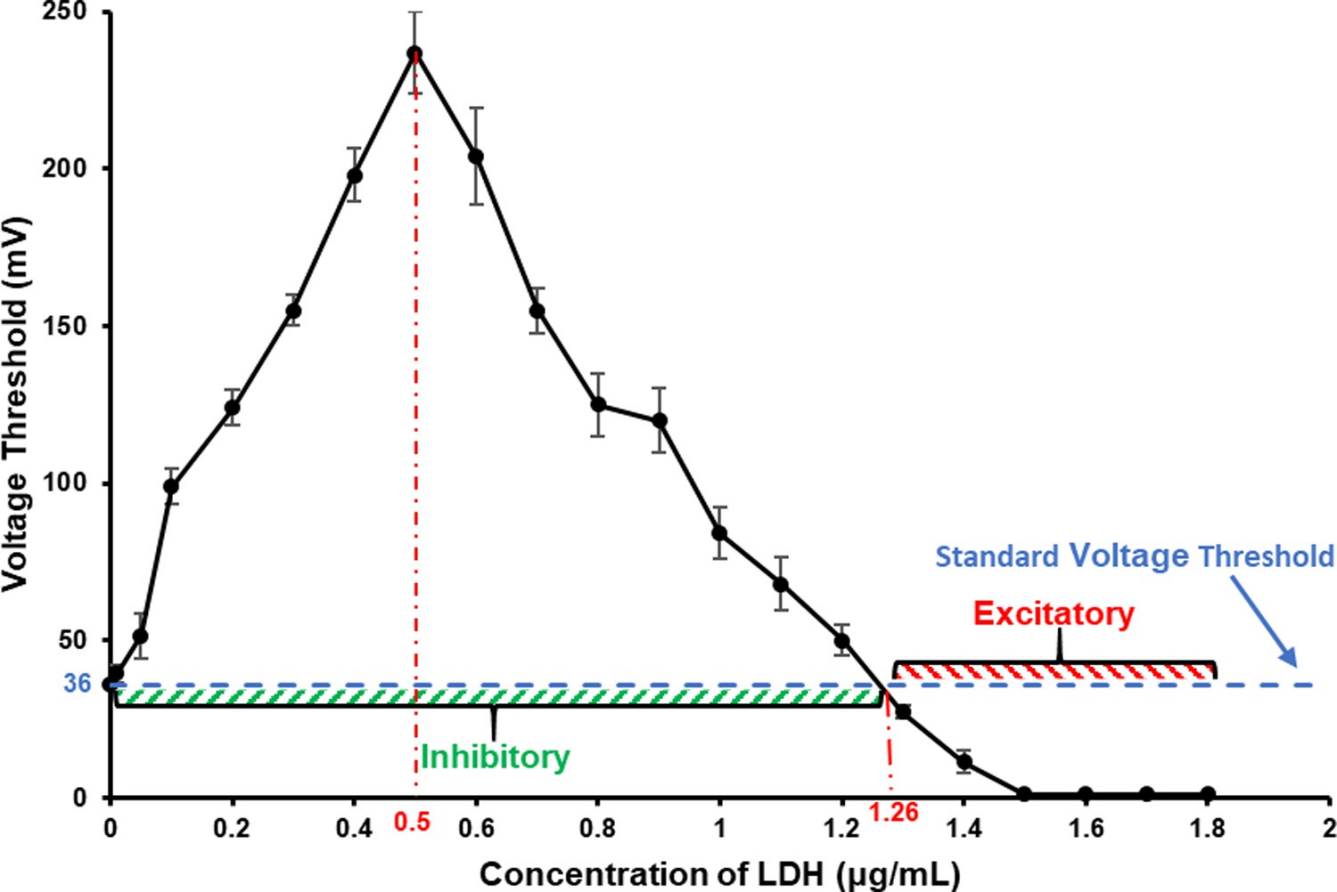
The *V*_Th_ of PC12 quasi-neuronal networks under the effect of LDH in different concentrations (*n* = 5). Blue dash line represents the normal *V*_Th_. The turning point of the effect of LDH from inhibitory to excitatory was at about 1.26 μg/mL which was calculated by linear fitting the two data points next to the turning point in the experiment.

In the research of the effect of LDH on PC12 quasi-neuronal networks, we found the changes of the *V*_Th_ of PC12 quasi-neuronal networks with LDH in different concentrations had a biphasic feature and showed a Λ-shaped curve (Fig 6, S4 Table). The *V*_Th_ of PC12 quasi-neuronal networks was higher than the normal *V*_Th_ when the LDH concentration was between 0.01–1.2 μg/mL, and LDH exhibited inhibitory effect. In the concentration range mentioned above, the *V*_Th_ of PC12 quasi-neuronal networks first went up with the increase of LDH concentration. While when the concentration of LDH went to 0.5 μg/mL, the *V*_Th_ reached the peak value and then went down with the increase of LDH concentration, which showed that LDH had the strongest inhibitory effect on PC12 quasi-neuronal networks at 0.5 μg/mL and the inhibitory effect weakened if continuously increase the LDH concentration. When the concentration of LDH rose up to ≥ 1.3 μg/mL, the *V*_Th_ of PC12 quasi-neuronal networks became lower than the normal *V*_Th_, which showed that LDH exhibited excitatory effect. When the concentration of LDH increased to 1.5 μg/mL, the *V*_Th_ of PC12 quasi-neuronal networks went down to 1 mV which was already the lowest output of the signal generator, while further increasing the LDH concentration to 1.8 μg/mL did not change the *V*_Th_. This showed when the concentration of LDH was ≥ 1.5 μg/mL, PC12 quasi-neuronal networks stayed in a highly excitatory status.

### Comparing the changes of the *V*_Th_ of PC12 quasi-neuronal networks with that of hippocampal neuronal networks and hippocampal slices under the effect of the four factors

Our group have studied the effects of ACh, ethanol, temperature and LDH on hippocampal neuronal networks and hippocampal slices through VTMM [6, 17]. To investigate whether PC12 quasi-neuronal networks have similar *V*_Th_ features with normal neuronal networks, we compared the effect of the four factors (ACh, ethanol, temperature and LDH) on the changes of *V*_Th_ of PC12 quasi-neuronal networks with that of hippocampal neuronal networks and hippocampal slices.

**Fig 7:**
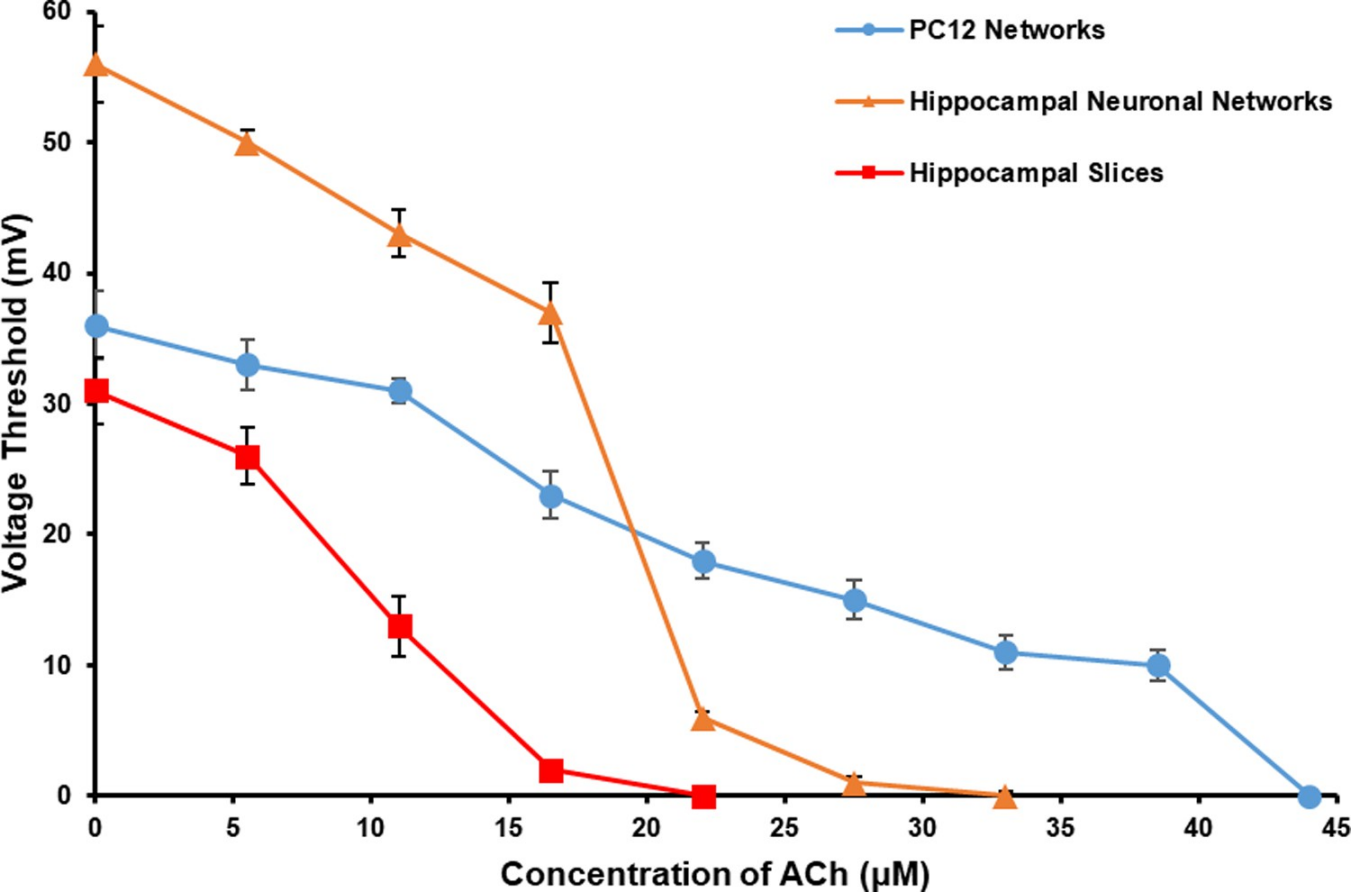
The *V*_Th_ of the three types of neuronal networks under the effect of ACh in different concentrations (*n* = 5).

As shown in Fig 7, the changes of *V*_Th_ of PC12 quasi-neuronal networks under the effect of ACh were similar with that of hippocampal neuronal networks and hippocampal slices, which decreased with the increase of ACh concentration. This illustrated the electrical excitability of PC12 quasi-neuronal networks went up with the increase of applied ACh concentration, and it was same as the results we observed from hippocampal neuronal networks and hippocampal slices.

**Fig 8:**
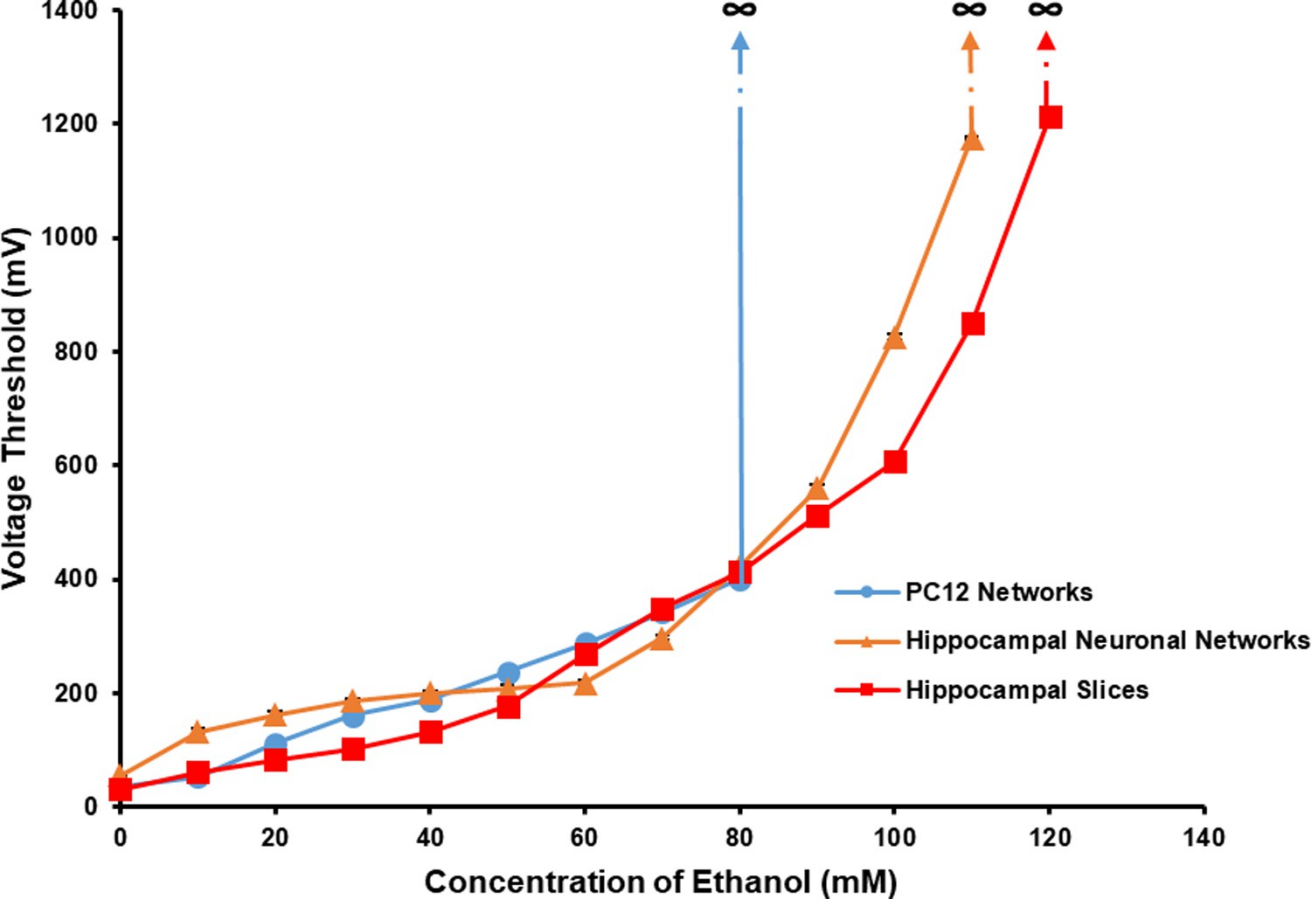
The *V*_Th_ of the three types of neuronal networks under the effect of ethanol in different concentrations (*n* = 5).

The changes of *V*_Th_ of the three types of neuronal networks under the effect of ethanol in different concentrations were shown in Fig 8. The three types of neuronal networks presented similar changing trends with the effect of ethanol and all showed the increasing *V*_Th_ with the increase of ethanol concentration. The three curves were especially uniform when the ethanol concentration was lower than 80 mM. The results demonstrated that the *V*_Th_ of PC12 quasi-neuronal networks had very similar changes with that of hippocampal neuronal networks and hippocampal slices under the influence of ethanol.

**Fig 9:**
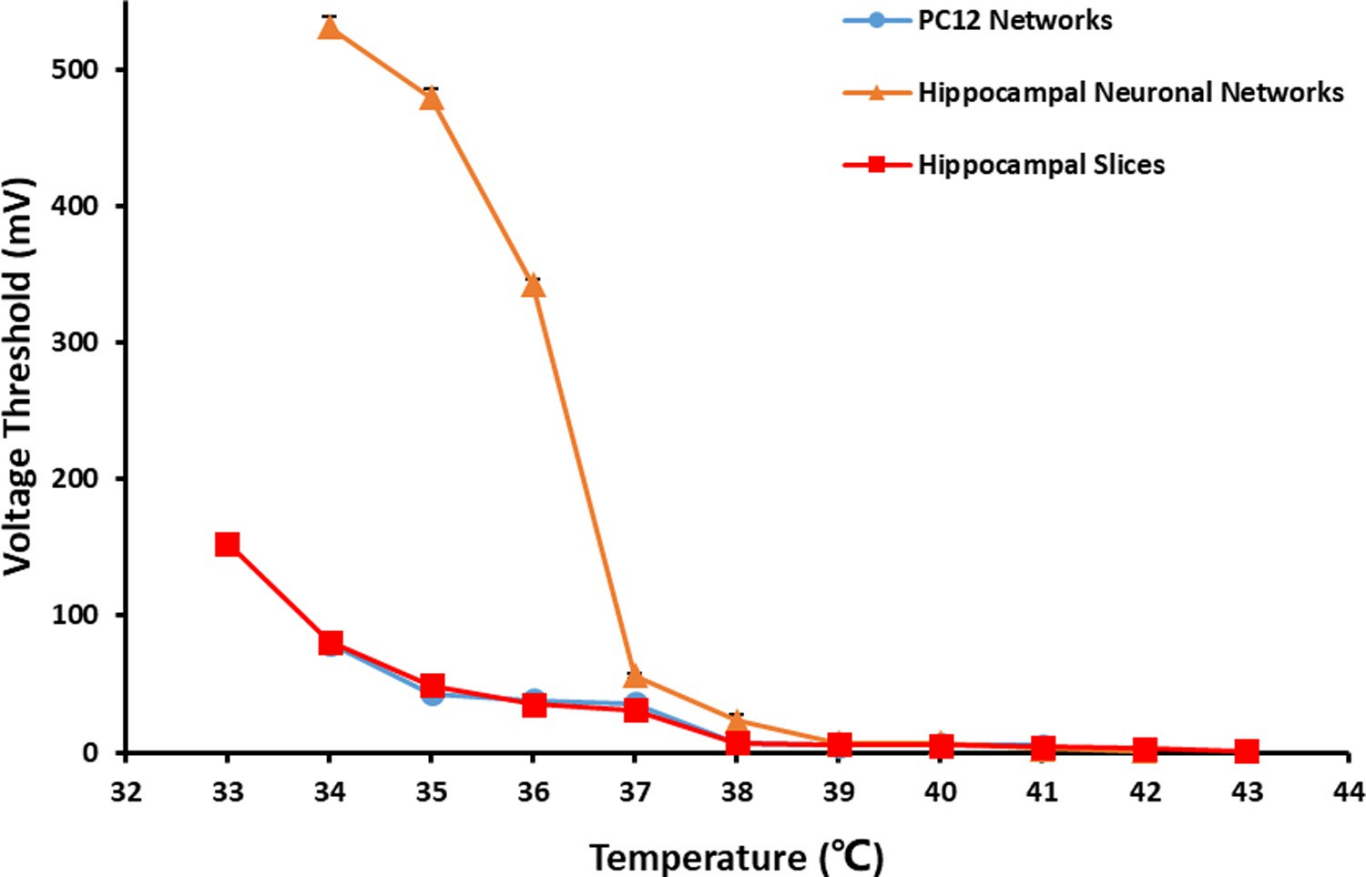
The changes of *V*_Th_ of the three types of neuronal networks with temperature (*n* = 5).

The changes of the three types of neuronal networks with temperature were shown in Fig 9. The changes of *V*_Th_ with temperature of them all presented as U-shaped curves. Under the temperature of 37°C, the normal *V*_Th_ of PC12 quasi-neuronal networks, hippocampal neuronal networks and hippocampal slices were different, which were 36, 56 and 31 mV, respectively. However, the experiment results showed under the effect of different temperature, the changes of *V*_Th_ of PC12 quasi-neuronal networks were similar with that of hippocampal neuronal networks and hippocampal slices.

**Fig 10:**
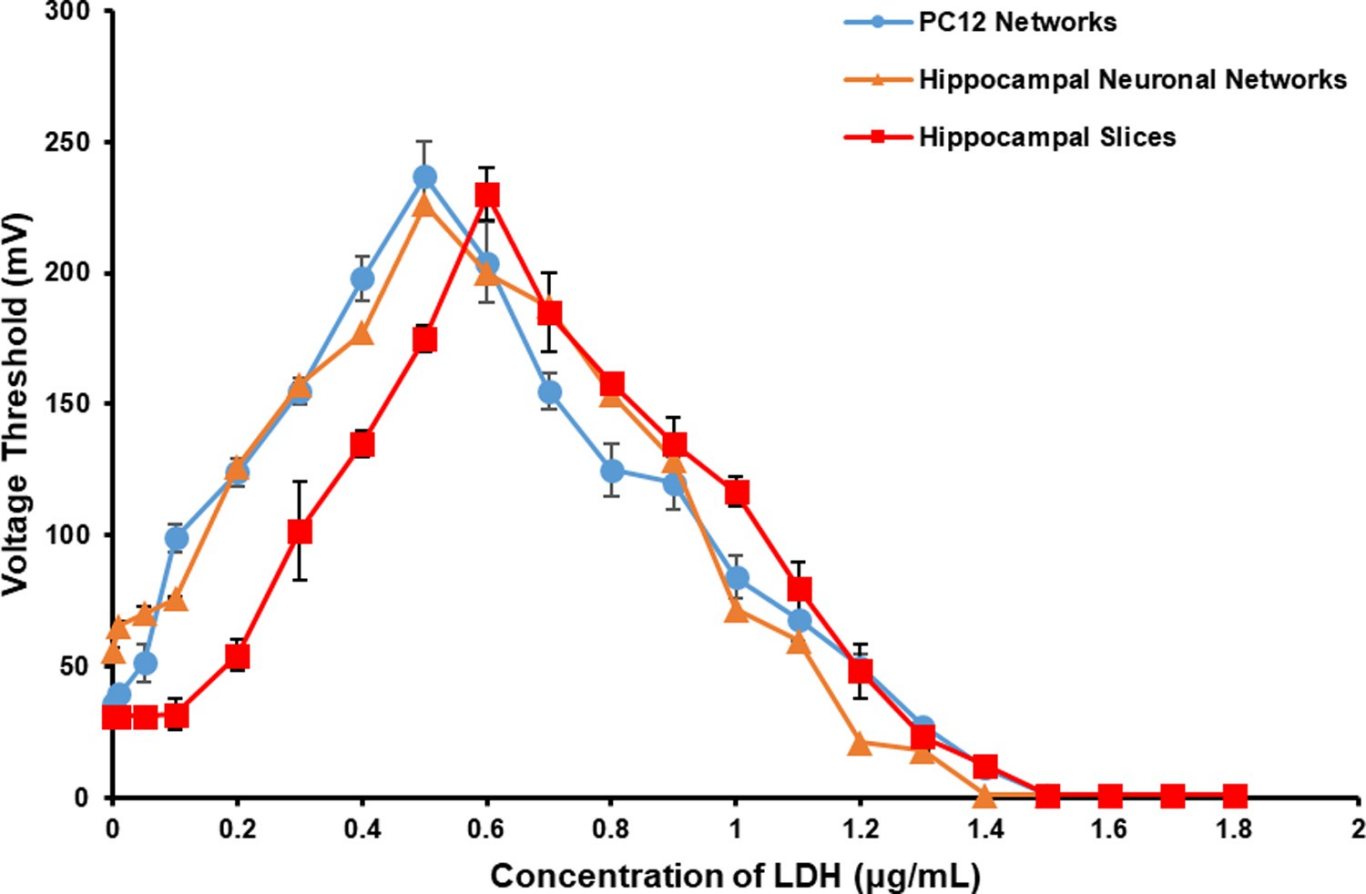
The *V*_Th_ of the three types of neuronal networks under the effect of LDH in different concentrations (*n* = 5 for PC12 networks, *n* = 4 for hippocampal neuronal networks and hippocampal slices).

The changes of *V*_Th_ of the three types of neuronal networks under the effect of LDH in different concentration were shown in Fig 10. LDH had very similar concentration-dependent effects on all the three networks. To be specific, low concentration of LDH had an inhibitory effect on the three types of neuronal networks and the effect strengthened with the increase of concentration until reached its peak, and then the inhibitory effect began to weaken with the increase of LDH concentration. While high concentration of LDH had an excitatory effect on all the three types of neuronal networks. The excitatory effect became stronger with the increase of LDH concentration and would finally maintain at a high level.

## Discussion

By comparing the effects of ACh, ethanol, temperature and LDH on the three types of neuronal networks, we found the reactions of PC12 quasi-neuronal networks to the applied factors had a lot of similarities with that of the other two neuronal networks, and the changes of their *V*_Th_ shared the same trend.

In the four factors, ethanol and temperature can influence multiple factors of neurons. Ethanol can influence the synthesis and transmission of neurotransmitters, and the generation, transport, and process of neural signals. It also can lead to cellular damage of neurons [18–22]. Benson DM et al. (1989) found that ethanol can obviously inhibit the electrical activities of neurons in the concentration from 50–100 mM [18]. And Xia Y et al. (2003) also reported that 20-160-mM ethanol could reversibly inhibit the electrical excitability of neuronal networks [19]. The results of our experiments agreed with these studies. The change of temperature can alter the electrophysiological characteristics of neurons. High temperature can increase the exchange rate of ions, influence the activities of different types of ion channels, and change the depolarization speed of neurons, while low temperature can slow down the metabolic rate, influence the release of neurotransmitters, inhibit the activities of ion pumps and receptors, and change the frequency of generation and transmission of neural signals [23–28]. Griffin JD et al. (1996) reported in the temperature range from 32–39°C, the increase of temperature could increase the discharge frequency of warm-sensitive neurons [24]. Burgoon PW et al. (2001) also found in the temperature range from 32–40°C, increasing the temperature could accelerate the depolarization process and elevate the discharging frequency of warm-sensitive neurons [25]. These conclusions also agreed with the results of our experiments. The *V*_Th_ of PC12 quasi-neuronal networks have similar reactions to ethanal and temperature with that of the other two neuronal networks, which proved the excitability of PC12 quasi-neuronal networks is basically similar with that of normal neuronal networks, and they all could be influenced by the factors mentioned above.

Comparing with ethanol and temperature, the influence of ACh and LDH focused on certain types of receptors on the surface of neurons. ACh is an important excitatory neurotransmitter which can modulate neurons through ACh receptors. PC12 cells and some types of neurons in hippocampal area are provided with ACh receptors [11, 12, 29, 30]. Thus, the reactions of the three types of neuronal networks to ACh were almost the same in the experiments. As a commonly used anesthetic, LDH can act on TRPV1 and GABA receptors and further change the electrical excitability of neurons [31–34]. PC12 cells were also equipped with TRPV1 and GABA receptors [35–37]. The similarity between the effects of LDH on the electrical excitability of PC12 quasi-neuronal networks and that of the other two types of neuronal networks were highly probably caused by that they shared same types of receptors.

The similarities of the effects of the four factors on the three types of networks suggested that under proper conditions, PC12 quasi-neuronal networks have strong potential for being employed as a simple replacement for normal neuronal networks in the studies of the influence of external factors such as neurotransmitters or blockers on the electrical excitability of neuronal networks. However, although the reactions of PC12 quasi-neuronal networks to the four external factors shared similar trends with normal neuronal networks, they were still different in details. This was caused by the differences between PC12 networks and normal neuronal networks. In PC12 quasi-neuronal networks there is only one type of cells, and ACh is the only neurotransmitter which could effectively activate the networks [11, 12, 38]. While in normal neuronal networks the cell types are much more complicated and multiple types of neurotransmitters could be involved in the cross talks between neurons. And the functions of glia cells also cannot be ignored in normal neuronal networks. The simplicity of PC12 quasi-neuronal networks provides great advantages during study, but it also limits the ability of PC12 quasi-neuronal networks to fully simulate normal neuronal networks. During experiments, the influence of all these factors must be considered before using PC12 quasi-neuronal networks as a substitute for normal neuronal networks.

## Conclusion

In this paper, VTMM was used to test the effects of ACh, ethanol, temperature and LDH on PC12 quasi-neuronal networks, and the results were further compared with the experiments completed earlier by our group on hippocampal neuronal networks and hippocampal slices. This is the first time that the electrical excitability of PC12 quasi-neuronal networks and that of other normal neuronal networks are compared under the same influential factors. The study proved that under the effects of the four factors, PC12 quasi-neuronal networks have similar *V*_Th_ changing features with that of the other two types of neuronal networks. PC12 is an established cell line, and PC12 cells have a rapid network formation speed and a much shorter culture cycle than primary cultured neurons. The experiment procedure to build PC12 quasi-neuronal networks is also much easier and more convenient than to build normal neuronal networks by acquiring primary neurons from animals or making brain slices. Using PC12 quasi-neuronal networks as a simple neuronal network model also can help to reduce the sacrifice of animals and is agree with the ethical requirement. Considering these advantages above, under proper conditions, PC12 quasi-neuronal networks are recommended to be employed as a simple neuronal network model for replacing primary cultured neuronal networks or brain slices in related research to study the electrical excitability of neuronal networks.

## Supporting information

S1 Table The *V*_Th_ of PC12 quasi-neuronal networks under the effect of ACh (*n* = 5). (DOCX)

S2 Table The *V*_Th_ of PC12 quasi-neuronal networks under the effect of ethanol (*n* = 5). (DOCX)

S3 Table The *V*_Th_ of PC12 quasi-neuronal networks under different temperature (*n* = 5). (DOCX)

S4 Table The *V*_Th_ of PC12 quasi-neuronal networks under different concentrations of LDH (*n* = 5). (DOCX)
