# Supplementary material for: Study on influence of external factors on the electrical excitability of PC12 quasi-neuronal networks through Voltage Threshold Measurement Method
Source: PLoS One. 2022 Mar 9;17(3):e0265078. doi: 10.1371/journal.pone.0265078 (PMC8906582; doi:10.1371/journal.pone.0265078)
Supplement: S1 Table — (DOCX) [file pone.0265078.s001.docx]

**S1 Table. The *V*_Th_ of PC12 quasi-neuronal networks under the effect of ACh (*n*=5)**

| ***C*_ACh_ (μM)** | 1 | 2 | 3 | 4 | 5 | *‾X*±SD (mV) |
| --- | --- | --- | --- | --- | --- | --- |
| 0 | 40 | 33 | 35 | 37 | 35 | 36±2.6 |
| 5.5 | 33 | 35 | 34 | 30 | 32 | 33±1.9 |
| 11 | 30 | 30 | 31 | 32 | 30 | 31±0.9 |
| 16.5 | 23 | 23 | 25 | 22 | 20 | 23±1.8 |
| 22 | 18 | 18 | 16 | 17 | 20 | 18±1.5 |
| 27.5 | 15 | 15 | 16 | 15 | 12 | 15±1.5 |
| 33 | 12 | 12 | 10 | 13 | 10 | 11±1.3 |
| 38.5 | 11 | 11 | 10 | 10 | 8 | 10±1.2 |
| 44 | 0 | 0 | 0 | 1 | 1 | 0±0.5 |
