# Supplementary material for: Study on influence of external factors on the electrical excitability of PC12 quasi-neuronal networks through Voltage Threshold Measurement Method
Source: PLoS One. 2022 Mar 9;17(3):e0265078. doi: 10.1371/journal.pone.0265078 (PMC8906582; doi:10.1371/journal.pone.0265078)
Supplement: S2 Table — (DOCX) [file pone.0265078.s002.docx]

**S2 Table. The *V*_Th_ of PC12 quasi-neuronal networks under the effect of ethanol (*n*=5)**

| ***C*_EtOH_ (mM)** | 1 | 2 | 3 | 4 | 5 | *‾X*±SD (mV) |
| --- | --- | --- | --- | --- | --- | --- |
| 0 | 40 | 33 | 35 | 36 | 35 | 36±2.6 |
| 10 | 53 | 55 | 50 | 53 | 52 | 53±1.8 |
| 20 | 110 | 115 | 110 | 112 | 112 | 112±2.0 |
| 30 | 162 | 165 | 160 | 160 | 160 | 161±2.1 |
| 40 | 188 | 185 | 190 | 190 | 185 | 188±2.5 |
| 50 | 239 | 240 | 238 | 240 | 235 | 238±2.1 |
| 60 | 289 | 289 | 290 | 288 | 285 | 288±1.9 |
| 70 | 341 | 341 | 342 | 342 | 345 | 342±1.6 |
| 80 | 399 | 399 | 400 | 400 | 405 | 401±2.5 |
| 90 | ∞ | ∞ | ∞ | ∞ | ∞ | ∞ |
