# Supplementary material for: Study on influence of external factors on the electrical excitability of PC12 quasi-neuronal networks through Voltage Threshold Measurement Method
Source: PLoS One. 2022 Mar 9;17(3):e0265078. doi: 10.1371/journal.pone.0265078 (PMC8906582; doi:10.1371/journal.pone.0265078)
Supplement: S3 Table — (DOCX) [file pone.0265078.s003.docx]

**S3 Table. The *V*_Th_ of PC12 quasi-neuronal networks under different temperature (*n*=5)**

| ***T*(°C)** | 1 | 2 | 3 | 4 | 5 | *‾X*±SD (mV) |
| --- | --- | --- | --- | --- | --- | --- |
| 33 | ∞ | ∞ | ∞ | ∞ | ∞ | ∞ |
| 34 | 80 | 75 | 80 | 85 | 80 | 80±3.5 |
| 35 | 45 | 43 | 42 | 42 | 43 | 43±1.2 |
| 36 | 38 | 37 | 39 | 39 | 38 | 38±0.8 |
| 37 | 40 | 33 | 35 | 37 | 36 | 36±2.6 |
| 38 | 7 | 7 | 6 | 8 | 7 | 7±0.7 |
| 39 | 5 | 4 | 5 | 6 | 6 | 5±0.8 |
| 40 | 7 | 6 | 5 | 6 | 6 | 6±0.7 |
| 41 | 3 | 5 | 7 | 5 | 5 | 5±1.4 |
| 42 | ∞ | ∞ | ∞ | ∞ | ∞ | ∞ |
