# Supplementary material for: Study on influence of external factors on the electrical excitability of PC12 quasi-neuronal networks through Voltage Threshold Measurement Method
Source: PLoS One. 2022 Mar 9;17(3):e0265078. doi: 10.1371/journal.pone.0265078 (PMC8906582; doi:10.1371/journal.pone.0265078)
Supplement: S4 Table — (DOCX) [file pone.0265078.s004.docx]

**S4 Table. The *V*_Th_ of PC12 quasi-neuronal networks under different concentrations of LDH (*n*=5)**

| ***C*_LDH_ (μg/mL)** | 1 | 2 | 3 | 4 | 5 | *‾X*±SD (mV) |
| --- | --- | --- | --- | --- | --- | --- |
| 0.01 | 40 | 42 | 42 | 35 | 38 | 39.4±3.0 |
| 0.05 | 48 | 46 | 58 | 60 | 45 | 51.4±7.1 |
| 0.1 | 100 | 105 | 90 | 100 | 100 | 99±5.5 |
| 0.2 | 120 | 130 | 120 | 130 | 120 | 124±5.5 |
| 0.3 | 160 | 150 | 160 | 155 | 150 | 155±5.0 |
| 0.4 | 190 | 190 | 210 | 200 | 200 | 198±8.4 |
| 0.5 | 250 | 250 | 265 | 280 | 230 | 237±13.0 |
| 0.6 | 200 | 190 | 200 | 230 | 200 | 204±15.2 |
| 0.7 | 150 | 150 | 165 | 150 | 160 | 155±7.1 |
| 0.8 | 140 | 115 | 120 | 130 | 120 | 125±10 |
| 0.9 | 110 | 120 | 130 | 120 | 120 | 120±10 |
| 1.0 | 85 | 90 | 70 | 85 | 90 | 84±8.2 |
| 1.1 | 70 | 80 | 60 | 70 | 60 | 68±8.4 |
| 1.2 | 50 | 45 | 55 | 55 | 45 | 50±5.0 |
| 1.3 | 25 | 30 | 25 | 28 | 28 | 27.2±2.2 |
| 1.4 | 15 | 10 | 15 | 10 | 7 | 11.4±3.5 |
| 1.5 | 1 | 1 | 1 | 1 | 1 | 1±0 |
| 1.6 | 1 | 1 | 1 | 1 | 1 | 1±0 |
| 1.7 | 1 | 1 | 1 | 1 | 1 | 1±0 |
| 1.8 | 1 | 1 | 1 | 1 | 1 | 1±0 |
